# The Effects of Soft-Tissue Techniques and Exercise in the Treatment of Patellar Tendinopathy—Systematic Review and Meta-Analysis

**DOI:** 10.3390/healthcare12040427

**Published:** 2024-02-07

**Authors:** Federico Ragone, Silvia Pérez-Guillén, Andoni Carrasco-Uribarren, Sara Cabanillas-Barea, Luis Ceballos-Laita, Pere Ramón Rodríguez-Rubio, Rosa Cabanas-Valdés

**Affiliations:** 1 Faculty of Medicine and Health Sciences, Universitat Internacional de Catalunya, 08195 Sant Cugat del Vallés, Spain; f.ragone99@uic.es (F.R.); acarrasco@uic.es (A.C.-U.); scabanillas@uic.es (S.C.-B.); prodriguez@uic.es (P.R.R.-R.); rosacabanas@uic.es (R.C.-V.); 2 Department of Surgery, Ophthalmology, Otorhinolaryngology and Physiotherapy, University of Valladolid, 42004 Soria, Spain; luis.ceballos@uva.es

**Keywords:** soft-tissue therapy, patellar tendinopathy, exercise therapy

## Abstract

Background: Patellar tendinopathy is a degenerative clinical disorder that causes load-related pain in the lower pole of the patella or patellar tendon. It predominantly affects young male athletes engaged in sports involving repetitive tendon loading, particularly explosive jumping. The combination of manual techniques with therapeutic exercise is hypothesized to provide greater benefits than exercise alone. Objective: The aim of this study is to analyze the scientific evidence regarding the effects of soft-tissue techniques combined with therapeutic exercise versus therapeutic exercise alone on pain intensity and function in individuals with patellar tendinopathy. Methods: A systematic review with meta-analysis was conducted following the PRISMA guidelines. PubMed, Lilacs, IBECS, CENTRAL, WOS, SciELO, Academic Search, CINAHL, SportDiscus, PEDro, and Google Scholar databases were consulted. Randomized controlled trials and quasi-randomized trials focusing on the effects of soft-tissue techniques combined with therapeutic exercise (experimental group) versus therapeutic exercise alone (control group) on pain and function in individuals aged 16 years and older with patellar tendinopathy were selected. The Cochrane tool for risk-of-bias assessment and the PEDro scale for methodological quality were used. Results and Discussion: A total of six studies (*n* = 309; age range = 16–40 years), considered to have a low risk of bias and moderate-to-high methodological quality, were included. The results showed improvements in function in the experimental group (mean of 60% on the Visa-P scale) and pain in the experimental group (mean decrease of 2 points in the VAS scale). There were improvements in 50% of the studies when comparing variables between the experimental and control groups. Conclusions: The combination of manual techniques, such as dry needling, percutaneous electrolysis, transverse friction massage, and stretching, along with a squat on a 25° inclined plane, appears to be effective in the treatment of patellar tendinopathy. Static stretching of the quadriceps before and after the squat five times per week, along with dry needling or percutaneous electrolysis sessions twice a week for 8 weeks, is recommended. However, future studies analyzing groups with passive techniques versus therapeutic exercise are needed to standardize the treatment and establish the optimal dose.

## 1. Introduction

Patellar tendinopathy (PT) is a clinical degenerative condition that causes load-related pain in the inferior pole of the patella or patellar tendon [1,2]. It predominantly affects young male athletes practicing sports that involve repetitive loading of the tendon, particularly explosive jumping [3]. The prevalence varies according to the sport played, being more common in elite and recreational volleyball and basketball. It is estimated that as many as 45% and 32% of these athletes, respectively, suffer from PT [3,4,5,6]. The recovery rates for PT are not satisfactory, causing significant time off sports and high recurrence rates [7,8,9].

The term “tendinitis” has been replaced by “tendinopathy”, since the minimal presence of inflammatory cells has been demonstrated, and histopathological studies confirm structural degenerative changes in the tendon tissue as the key feature of tendon dysfunction [1,10]. Thus, tendon pathology is described as a continuum of three tissue states: reactive tendinopathy, unstructured tendon, and degenerative tendinopathy. The most widely accepted etiological factors are mechanical overload and repetitive microtrauma, although other intrinsic and extrinsic factors may also be predisposing factors for pathology [9,11]. Some risk factors include hamstring and quadriceps muscle tightness, reduced ankle dorsiflexion, foot hyperpronation, poor joint coordination, overweight, leg length discrepancy, an increased volume and intensity of jump training, and activity on hard courts and synthetic turf [12]. Many of these risk factors are thus modifiable and preventable through appropriate physiotherapy programs [12,13,14]. A diagnosis of PT is typically based on the clinical history and symptomatic findings, with color-Doppler ultrasound (CD-US) and magnetic resonance imaging (MRI) being the most used methods to confirm tendon pathology [15].

The management of PT can be active or passive. Active strategies involve tendon-loading regimes, and eccentric training is the most widely adopted approach. It has been shown that there is a 50–70% likelihood of improvement at 3–6-month follow-up with this method [14,16]. In fact, many authors have advocated for eccentric training as the gold standard in the treatment of tendinopathies [17,18,19]. Passive treatments for PT include different minimally invasive techniques (MITs), such as corticosteroid and platelet-rich plasma (PRP) injections, extracorporeal shockwave therapy (ESWT), low-energy laser therapy, dry needling (DN), and percutaneous needle electrolysis (PNE) [20,21,22].

The conclusion of the systematic review of Everhart JS et al. in 2017 was that the initial treatment of PT may consist of eccentric exercise, shockwave, or PRP as monotherapy or an adjunct to accelerate recovery. Surgery or ESWT should be considered if the conservative treatment fails after 6 months [23]. A recent meta-analysis determined that MIT, when combined with exercise, was effective post-treatment and at follow-up [21]. Several authors have also studied the effects of manual non-invasive soft-tissue techniques, such as stretching, massage, and muscle tissue mobilization, showing a reduction in pain and an improvement in the function of patients with PT. It seems that these techniques have different theoretical mechanisms of action but the same aim of restoring the normal healing of the affected tissues and muscle imbalances [24,25,26].

To the best of our knowledge, within MIT, there are some procedures that are outside the scope of competence of physiotherapy, such as PRP or corticosteroid injection. However, DN and PRP are two MITs that, together with manual non-invasive soft-tissue techniques, constitute therapeutic strategies to address PT. We also believe that the use of standardized symptom scores, such as the Victorian Institute of Sports Assessment Patellar Tendinopathy Questionnaire (VISA-P), in the current PT literature allows for a more quantitative assessment of treatment outcomes using statistical techniques to gain new insight into the efficacy of these treatments [26,27]. Consequently, the aim of this systematic review and meta-analysis is to evaluate the effectiveness of incorporating soft-tissue techniques into exercise therapy in improving pain and function in patients with PT. It is hypothesized that the combination of any of the previously mentioned muscle techniques with exercise therapy may provide better results in pain and function than exercise therapy alone in patients with PT.

## 2. Materials and Methods

### 2.1. Study Design

A systematic review and meta-analysis were conducted following the Preferred Reporting Items for Systematic Review (PRISMA) [28] guidelines to investigate the effectiveness of soft-tissue techniques in patients with patellar tendinopathy. The review was registered in the international prospective register for systematic reviews (PROSPERO) database (CRD42022501825).

### 2.2. Data Sources and Search

The following computerized databases were consulted by two independent reviewers (FR and SP) to carry out the present review: Pubmed, Lilacs, IBECS, CENTRAL, WOS Core Collection, SciELO, Academic Search, CINAHL, SportDiscus, PEDro, and Google Scholar. The search strategy involved a combination of MeSH terms and free terms, such as “Patellar Ligament”, “Exercise Therapy”, “Musculoskeletal Manipulations”, “Acupuncture Therapy”, “Muscle Stretching Exercises”, and “Electrolysis”, with the truncation term “Random*” to locate randomized clinical trials. The search was completed on the 10th of April 2023. The adapted search strategies for each database are included in Supplementary Material S1.

### 2.3. Study Selection Process and Eligibility Criteria

The article selection process, identified through the literature search and the removal of duplicate findings in databases, was carried out using the RayyanQCRI software (1 Broadway Street, 14th Floor, Cambridge, MA 02142, USA). The selection process was performed by two independent reviewers (FR and SP) in two stages. After identifying all the studies, a first screening was carried out by reading the titles and abstracts. From the selected articles, a full-text reading was conducted to verify whether they met the inclusion or exclusion criteria. Any discrepancies were solved by a third reviewer (RC) if necessary.

Only studies that met the following inclusion criteria were included: (P: population) participants of all sexes and ages =/> 16 years diagnosed with patellar tendinopathy in any stage of the disease; (I: intervention) studies that evaluated the effectiveness of soft-tissue techniques combined with therapeutic exercises in the affected knee region, (C: comparison) compared to the application of therapeutic exercise alone; (O: outcomes) studies that evaluated one of the following variables: pain intensity and tendon function; (S: studies) Randomized and quasi-randomized controlled trials published in national and international journals.

As part of the selection criteria, the included articles were rated using the Physiotherapy Evidence Database (PEDro) scale. Studies with scores between 6 and 8 are classified as having good methodological quality, scores between 4 and 5 indicate regular quality, and scores below 4 points are considered to have poor methodological quality [29].

### 2.4. Data Extraction

Data extraction was performed in pairs according to the P.I.C.O. strategy. Data were extracted on participant characteristics (sample size, age, sex, disease severity), intervention and control group characteristics (type of intervention, type of soft tissue addressed, intensity, frequency, duration of session and program), outcome variables on *Victorian Institute of Sport Assessment-Patella (Visa-P*) and *Visual Analog Scores* (*VAS*), and the type of study design.

### 2.5. Risk-of-Bias and Methodological Quality Assessment

The methodological quality was assessed using the PEDro scale [30] by two blinded evaluators (FR and SP), and discrepancies were solved by a third reviewer (RC). This tool consists of a total of 11 items that assess external validity, internal validity, and statistical analysis. The first item of the PEDro scale was not considered in this review. Thus, the maximum score for an article did not exceed 10 points, with the minimum score being 0 points.

The same blinded evaluator (FR and SP) performed the assessment of the risk of bias. The Cochrane Collaboration tool was used for this aim [31]. This tool evaluates six domains, including sequence generation (selection bias), allocation concealment (selection bias), the blinding of participants and personnel (performance bias), the blinding of outcome assessment (detection bias), incomplete outcome data (attrition bias), selective reporting (reporting bias), and other biases. Each item is scored as “high risk”, “low risk”, or “unclear risk”.

### 2.6. Strength of Evidence

The quality of the evidence was assessed through the Grading of Recommendations, Assessment, Development, and Evaluation system (GRADE) [32]. The GRADE system evaluates the quality of evidence based on the extent to which users can be confident that the reported effect reflects the element being evaluated.

The assessment of the quality of evidence includes the risk of bias in the studies, inconsistency, imprecision, publication bias, indirect results, and other factors that may influence the quality of evidence. To summarize this information, summary tables of the findings were developed.

### 2.7. Statistical Analysis

A random-effects meta-analysis was performed using the RevMan version 5.4 software (The Cochrane Collaboration, Copenhagen, Denmark). The mean difference (MD) was used if all studies used the same tool to measure an outcome, and a standard mean difference (SMD) was used if the tool varied between studies. The generic inverse variance method was used for adjusted effect estimates and its standard error. Each study estimate of the relative treatment was given a weight that is equal to the inverse of the variance of the effect estimate. We used a *p*-value of less than 0.05 to determine statistical significance. The effect size was categorized as 0.2, 0.5, 0.8, and 1.3, which were considered small, medium, large, and very large, respectively. All effect size measures were expressed with a 95% confidence interval. Heterogeneity was expressed and visually assessed by forest plots and using the I2 statistic. The I2 statistic describes the percentage of total variation across studies that is attributable to heterogeneity rather than chance. A value greater than 25% is considered to reflect low heterogeneity, 50% moderate, and 75% high heterogeneity.

A narrative review and tables were used when there was insufficient data for quantitative analysis. Missing data from studies was requested by email from the corresponding author.

## 3. Results

### 3.1. Study Selection

**Figure 1:**
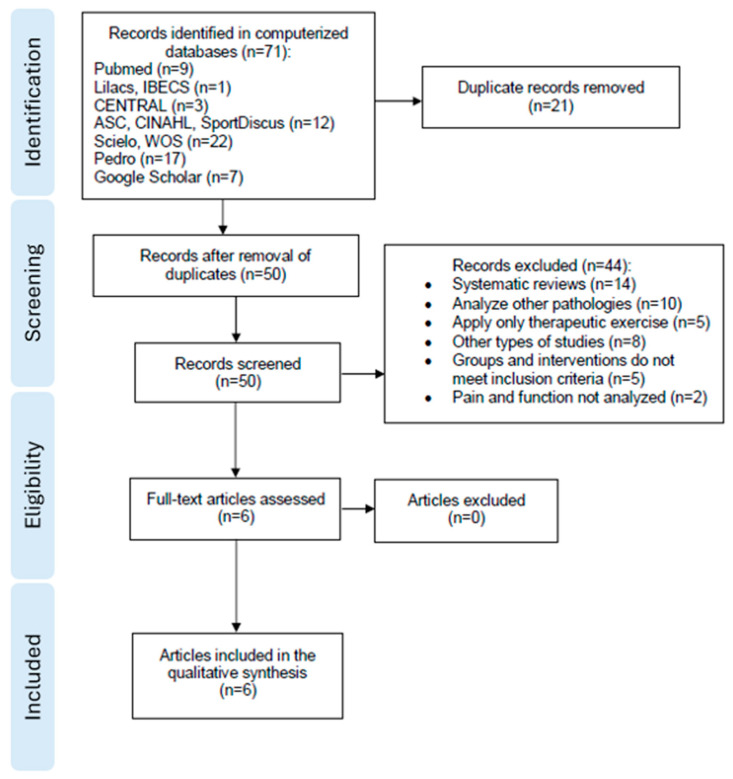
PRISMA diagram.

The PRISMA diagram (Figure 1) summarizes the results of the scientific literature search. Out of the total number of databases consulted, a total of 71 studies were obtained. After removing duplicates, the titles and abstracts of 50 studies were reviewed, and a total of 6 trials met the inclusion criteria. After reading the full text of these studies, no other articles were excluded. Finally, a total of 6 trials were included in this systematic review.

They were randomized and quasi-randomized controlled clinical trials (*n* = 6) published from 2012 to 2023. The studies were conducted in Greece [25], USA [33], India [24], Spain [21,34] and Pakistan [35].

### 3.2. Quality and Risk of Bias

Two independent reviewers analyzed and measured the methodological quality and risk of bias of the included studies using the PEDro scale [30] and The Cochrane Collaboration scale [32]. The methodological quality of the included studies ranged from 6 to 8 points, with an average of 6.83 points [6,7,8,9,10,11]. The homogeneity between the studies was quite good. All studies were assigned a “No” for not blinding the physiotherapists who administered the therapy, as well as the subjects included. Only Dragoo et al. [33] blinded both the therapists and the participants. Only Dimitrios et al. [25] obtained a negative score for not randomizing the participants correctly. The remaining authors obtained a positive score in the randomization of the participants, but Jadhav et al. [24] and Abat et al. [34] did not mention whether the assignment was concealed. There was a positive homogeneity between all studies in items 8, 9, 10, and 11, except for Abat et al. [34] and López-Royo et al. [21].

In the “generation of sequence” and “allocation concealment”, only Dimitrios et al. [25] obtained a high risk of bias in these two items. The highest risk of bias was found in “blinding of participants and personnel”, in which almost all studies had a high risk of bias. Only Dragoo et al.’s study [33] was assigned a low risk of bias because both the orthopedic surgeon and the patients remained blinded with respect to the treatment group. The majority of the studies had a low risk of bias in “incomplete outcome data” and “other biases”. In the “blinding of evaluators”, Dragoo et al. [33] and Jadhav et al. [24] had a high risk of bias, while Jadhav et al. [24] and López-Royo et al. [21] had a high risk of bias in “selective reporting” because the authors did not report all results according to the study protocol. Finally, a low risk was assigned to the other studies.

### 3.3. Participants

This systematic review included a total of 309 participants, with an average of 51.5 (±25.05) patients per study. The age range of the participants was 16–36 years old. The sample had a higher percentage of male participants (77%, *n* = 235) than female participants (23%, *n* = 70), except for two studies that only included male participants [24,33]. Notably, Dragoo et al. [33] had the smallest sample size with 22 participants, while Sharif et al. [35] had the largest sample size with 96 participants. All participants were diagnosed with patellar tendinopathy, with a Visa-p scale score ranging from 37 to 69.1 points, which quantifies the severity of the pathology. The authors agreed on the eligibility criteria for patients: being over 16 years old, feeling pain on palpation of the lower pole of the patella, having a clinical diagnosis of patellar tendinopathy, experiencing symptoms that persist for more than three months, and practicing sports [21,24,25,33,34,35]. There was also considerable homogeneity in the exclusion criteria, with previous surgery and local injections of corticosteroids or other drugs being the most commonly used [21,24,33,34,35]. Only Dimitrios et al. [25] did not mention the exclusion criteria.

### 3.4. Quality of Evidence

**Figure 2:**
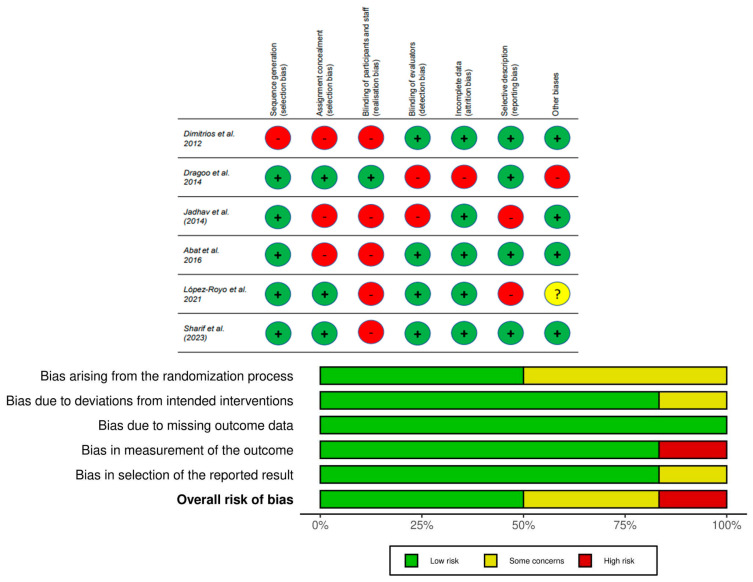
RoB [21,24,25,33,34,35].

The findings on the quality of evidence are summarized in Figure 2. Overall, the studies reviewed have a moderate level of quality, which implies that there is a possibility that the actual effect is far from the estimated effect.

For the six RCTs, “risk of bias” and “inconsistency” were assessed as “not serious”, because in most domains, the risk of bias is low, in addition to the fact that there is quite a high homogeneity among all results for the variables Visa-p and VAS. “Indirect evidence” was assessed as “not serious” and “imprecision” as “very serious”, because the included studies had a small sample size. The quality of evidence was downgraded by one point, in that the available evidence was based on few studies with positive results and, in some cases, was subject to funding. Conversely, it went up by one point because studies with higher doses/frequency of treatment were observed to show a greater response.

### 3.5. Characteristics of the Intervention

#### 3.5.1. Therapeutic Exercise

The authors reviewed the therapeutic exercise interventions used in the experimental and control groups across multiple studies and found that there was a high level of similarity among them. A protocol of exercises based on a single-leg or double-leg squat on a 25°-inclined board was applied, with a focus on the eccentric phase, meaning a greater duration of this phase. In addition, static stretching of the quadriceps and hamstrings was applied in one study [24], and conventional physiotherapy protocols were used in another study [35], which included stretching, strengthening exercises, ultrasound application, and transverse friction massage. The authors agreed on three sets of 15 repetitions with a rest period between 2 and 3 min. Patients were instructed to train at a perceived intensity of no pain, little pain, or a pain score of 5 out of 10 during eccentric exercises. However, Dragoo et al. [33] only mentioned that a three-phase eccentric exercise program (concentric–isometric–eccentric) would be applied to both groups without specifying the sets, repetitions, rest periods, intensity, or types of exercises provided (Supplementary Material S2).

#### 3.5.2. Soft-Tissue Techniques

Regarding the soft-tissue techniques and structures involved, all authors focused their interventions on the quadriceps, especially on the patellar tendon, which was the most treated structure. Three studies used instrumental techniques. Dragoo et al. [33] and Sharif et al. [35] used ultrasound-guided DN, while Abat et al. [34] used ultrasound-guided intratissue percutaneous electrolysis (EPI), and López-Royo et al. [21] used both techniques. However, despite differences in the intensity and dosage, there was a consensus on the location of the portion of the patellar tendon to be needled. Dimitrios et al. [25] administered static stretches of 30 s with a 1 min rest between each stretch before and after completing eccentric exercise. However, they did not describe the types of stretches or the number of sets of each one. Meanwhile, Jadhav et al. [24] applied 10 min of transverse massage to the intervention group and 10 min of cryotherapy to the control group.

#### 3.5.3. Frequency and Duration of Intervention

The frequency and duration of interventions varied among studies. Most studies prescribed eccentric exercises and stretching 2–5 times a week for 4–12 weeks, with one study prescribing twice daily exercises [21]. Studies using DN and EPI interventions included sessions every 2 weeks for 8 weeks.

### 3.6. Effectiveness of the Intervention

#### 3.6.1. Severity of Symptoms

The Visa-p scale was used to assess the symptoms of individuals.

**Figure 3:**
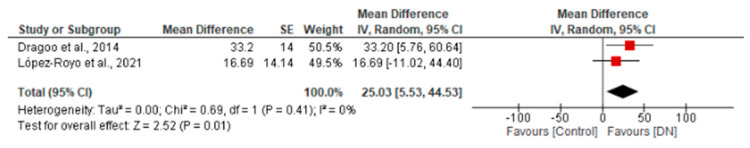
Forest plot of the Visa-p scale [21,33].

A meta-analysis was performed for DN (Figure 3). A pooled mean difference of 25.03 (95% CI from 5.53 to 44.53, *p* = 0.01) was observed, and no heterogeneity was shown. Dragoo et al.’s [33] measurements were taken at 26 weeks and Lopez-Royo et al.’s [21] at 10 weeks.

The results showed significant improvements in all experimental groups, with a range of 29% to 95% improvement in the first evaluations [21,25,34]. Only two studies did not show significant changes in the early evaluations [24,33]. However, by the later evaluations, there was an average improvement of 67% [21,24,25,33]. The study by Dimitrios et al. [25] reported a remarkable 113% improvement in the Visa-p score in week 24. In the Sharif et al. [35] study, the median and IQR were used instead of the mean and standard deviation, and significant improvements were observed in the Visa-p score in all evaluations in the DN group, but only in the first two evaluations in the control group.

The control groups in all studies also showed improvements in function, with an average improvement of 43% in the first evaluations, similar to the experimental groups. However, in the later evaluations, the average improvement dropped to 42% [21,24,25,33,34] compared to 67% in the experimental groups. Two studies added additional interventions to the control groups, but there was not much difference in the average improvement compared to the experimental groups that received DN or percutaneous electrolysis.

Regarding intergroup comparisons of the Visa-p scale, significant changes were reported in 50% of the studies, with the most significant improvement observed in the study by Dimitrios et al. [25] and Jadhav et al. [24]. Sharif et al. [35] reported significant changes in all evaluations, while Dragoo et al. [33] showed a negative change of 21% in favor of the control group.

#### 3.6.2. Pain Intensity

**Figure 4:**
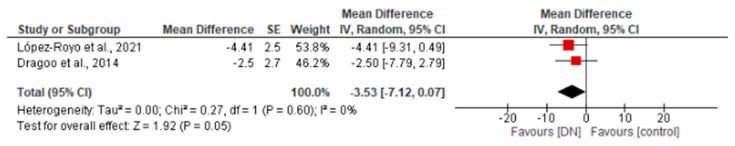
Forest plot of the pain intensity [21,33].

Pain intensity was assessed by the VAS. A meta-analysis was performed for DN (Figure 4). A pooled MD of −3.53 (95% CI from −7.12 to 0.07, *p* = 0.05) was obtained, and no heterogeneity was shown. For the meta-analysis, the data from Drago et al. [33] at 26 weeks and by López-Royo et al. [21] at 10 weeks were used.

According to Sharif et al. [35] in the comparison between DN and the control group, significant differences were observed in reducing pain in 2 and 4 weeks, suggesting that DN was more effective than conventional therapy. Jadhav et al. [24] reported only a 13% significant change in week 12, and no significant differences were found in the other articles [21,33].

#### 3.6.3. Quality of Life

**Figure 5:**
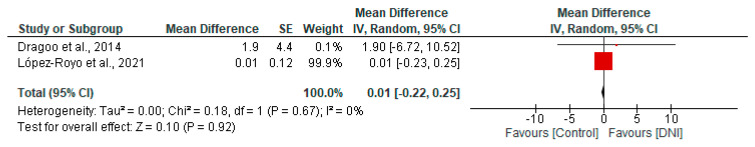
Forest plot of the quality of life [21,33].

Quality of life was assessed by SF-36 and SF-12. A meta-analysis was performed for DN (Figure 5). A pooled MD of 0.01 (95% CI from −0.22 to 0.25, *p* = 0.92) was obtained, and no heterogeneity was shown. No differences were revealed. Dragoo et al.’s [33] assessment was conducted at 26 weeks and Lopez-Royo et al.’s [21] at 10 weeks.

## 4. Discussion

This systematic review and meta-analysis assessed the effects of several muscle techniques when combined with exercise therapy on pain intensity and function in patients suffering from PT. Six RTCs were identified, and the results show a tendency toward a greater improvement in these two outcomes when exercise therapy was combined with muscle techniques versus when it was applied in isolation. A meta-analysis was conducted with two RCTs that included DN.

Manual therapy embraces a wide variety of muscle techniques for the treatment of tendinopathies, including manual and instrumental approaches. These techniques are directed to either the tendon, muscle belly, or fascial tissue. In this systematic review, a local approach directly addressing the patellar tendon structure prevailed. A broad body of literature has shown the correlation between quadriceps shortening and an increased risk of developing long-lasting PT [12,14,15], suggesting that the normalization of quadriceps length may lead to better recovery from this condition. In this line, Zhang ZJ et al. showed an increase in passive muscle tension in the vastus lateralis that is associated with proximal patellar tendon stiffness in athletes, suggesting that a muscle-specific approach is needed in the prevention and rehabilitation of PT [36]. According to fascial manipulation theory, patellar tendon pain is often due to an uncoordinated contraction of the quadriceps caused by abnormal fascial tension in the thigh. Therefore, the coordination deficit should be identified instead of directing attention solely to the patellar tendinopathy [37]. A broader approach addressing not only the patellar tendon but also other muscle structures, such as the quadriceps muscle and fascial tissue, may be useful for treating PT. This type of fascial approach has been shown to be effective in the treatment of lateral elbow tendinopathies when an instrumental technique by means of a hook is applied to muscle septa in the forearm [38].

The results of the meta-analysis seem to suggest that improvements in both pain and function are maintained at mid-term and 10 and 22 weeks after the first treatment with DN. This is a crucial fact for PT, as recovery rates for these patients are not satisfactory, with 49% of patients experiencing recurrent symptoms [39] and more than 50% of them retiring from sports participation due to persistent pain [7]. Authors have not reached a consensus on the underlying DN mechanisms, but they may be related to the release of cellular and humoral mediators, which promote the healing of tendon tissues [40].

Percutaneous needle electrolysis (PNE) [41,42] has shown excellent results in the short term, with a rapid return to the previous level of activity [43]. However, no structural changes in the tendon have been shown to be derived from DN or PNE [21]. This is an important fact to consider when performing the follow-up of patients with PT, as clinical findings may improve, but this is not necessarily accompanied by an improvement in the ultrasound image. Thus, return-to-play decisions for players with PT should be made based on functional outcomes, such as VISA-p scores, which include information about symptoms, functional tests, and subjects’ ability to perform sports activities and have been shown to be highly reproducible for both test–retest and inter-evaluator scoring (r > 0.95) [44].

Other less conservative treatments have proven their effectiveness in the treatment of PT. Injectable techniques, such as platelet-rich-plasma (PRP) infiltrations, manage to have long-term positive effects on PT. However, other treatments are more effective than PRP [33]. One such treatment is skin-derived tenocyte-like cells, which have been shown to be effective for recovery from other tendinopathies, such as lateral epicondylitis [45].

The methodological quality and risk of bias revealed a lack of blinding for subjects, therapists, and assessors. Only one trial documented blinding for either therapists or subjects. Blinding participants proves to be challenging, particularly in non-needle interventions, as conducting a reliable placebo-controlled experiment is often difficult. Additionally, blinding the therapist is unattainable in most muscle techniques, given that a portion of the treatment efficacy relies on the intensity and force applied by the physiotherapist.

To the best of our knowledge, this is the first systematic review and meta-analysis covering non-injectable muscle techniques. This is particularly relevant for the fields of physiotherapy and manual therapy, as injectable interventions are not within the scope of competency of the profession. These results may shed some light on conservative techniques that are very frequently used clinically when treating patients with PT. However, it has several limitations due to the inherent biases of the included studies. Thus, caution should be taken when interpreting the findings. First, there is a scarcity of RCTs concerning muscle techniques that do not rely on the infiltration of substances. This hinders the possibility of performing a meta-analysis with all the muscle techniques applied in the studies included in the systematic review. Second, the standardization of treatment may be necessary. Whereas exercise therapy applied in the studies was quite homogeneous in terms of the type of exercise and dosage, muscle techniques showed greater heterogeneity. Only six randomized controlled trials were included, and only two were meta-analyzed, which complicates the interpretation of the results, which should be interpreted with caution. Finally, only subjects over 16 years of age were included. We considered that subjects under that age could have confounding factors and that patellar tendinopathy may be related to growth factors. For this reason, the results of this meta-analysis cannot be extrapolated to that age group.

## 5. Conclusions

This systematic review and meta-analysis showed that the combination of manual techniques, such as DN, percutaneous electrolysis, transverse friction massage, and stretching, along with a squat on a 25°-inclined plane, appears to be effective in the treatment of patellar tendinopathy. Static stretching of the quadriceps before and after the squat five times per week, along with DN or percutaneous electrolysis sessions twice a week for 8 weeks, is recommended. But the results should be interpreted with caution, as only a few studies were meta-analyzed. Future studies analyzing groups with passive techniques versus eccentric exercises are needed to standardize the treatment and establish the optimal dose.

## Data Availability

The raw data supporting the conclusions of this article will be made available by the authors on request.

## Supplementary Materials

The following supporting information can be downloaded at https://www.mdpi.com/article/10.3390/healthcare12040427/s1. Supplementary Material S1: Search strategy. Supplementary Material S2: Characteristics of the intervention.
