# Supplementary material for: The Effects of Soft-Tissue Techniques and Exercise in the Treatment of Patellar Tendinopathy—Systematic Review and Meta-Analysis
Source: Healthcare (Basel). 2024 Feb 7;12(4):427. doi: 10.3390/healthcare12040427 (PMC10887760; doi:10.3390/healthcare12040427)
Supplement: Supplementary file 1 [file healthcare-12-00427-s001.zip › healthcare-2730263-supplementary S1.pdf]

Supplementary material S1. search strategies

Pubmed

("Patellar Ligament"[Mesh] AND "Exercise Therapy"[Mesh] AND ("Musculoskeletal Manipulations"[Mesh] OR "Acupuncture Therapy"[Mesh] OR "Muscle Stretching Exercises"[Mesh] OR "Electrolysis"[Mesh])) OR (("Patellar Ligament"[tw] OR "Patellar tendinopathy"[tw]) AND "Exercise Therapy"[tw] AND ("Musculoskeletal Manipulations"[tw] OR ("Acupuncture Therapy"[tw] OR "dry needling" [tw]) OR ("Muscle Stretching Exercises"[tw] OR stretching[tw])OR ("Electrolysis"[tw] OR EPI[tw] OR "intratissue percutaneous electrolysis" [tw]))) AND random*)

Lilacs, IBECS

mh:("Patellar Ligament" AND "Exercise Therapy" AND ("Musculoskeletal Manipulations" OR "Acupuncture Therapy" OR "Muscle Stretching Exercises" OR "Electrolysis")) OR Tw:(("Patellar Ligament" OR "Patellar tendinopathy") AND "Exercise Therapy" AND ("Musculoskeletal Manipulations" OR ("Acupuncture Therapy" OR "dry needling") OR ("Muscle Stretching Exercises" OR stretching)OR ("Electrolysis" OR EPI OR "intratissue percutaneous electrolysis")))

Cochrane Library

#1	("patellar ligament"):ti,ab,kw (Word variations have been searched)	334
#2	("exercise therapy"):ti,ab,kw (Word variations have been searched)	14652
#3	(manipulation):ti,ab,kw (Word variations have been searched)	14151
#4	("acupuncture"):ti,ab,kw (Word variations have been searched)	17655
#5	(stretching):ti,ab,kw (Word variations have been searched)	9244
#6	(electrolysis):ti,ab,kw (Word variations have been searched)	105
#7	MeSH descriptor: [Patellar Ligament] explode all trees	165
#8	MeSH descriptor: [Exercise Therapy] explode all trees	16556
#9	MeSH descriptor: [Musculoskeletal Manipulations] explode all trees	3396
#10	MeSH descriptor: [Acupuncture Therapy] explode all trees	5394
#11	MeSH descriptor: [Muscle Stretching Exercises] explode all trees	700
#12	MeSH descriptor: [Electrolysis] explode all trees	21

#13	(#1 AND #2 AND (#3 OR #4 OR #5 OR #6)) OR (#7 AND #8 AND (#9 OR #10 OR #11 OR #12)) 3

Academic Search Complete, CINAHL, SportDiscus (EBSCOHost)

(("Patellar Ligament" OR "Patellar tendinopathy") AND "Exercise Therapy" AND ("Musculoskeletal Manipulations" OR ("Acupuncture Therapy" OR "dry needling") OR ("Muscle Stretching Exercises" OR stretching)OR ("Electrolysis" OR EPI OR "intratissue percutaneous electrolysis"))) OR MH("Patellar Ligament" AND "Exercise Therapy" AND ("Musculoskeletal Manipulations" OR "Acupuncture Therapy" OR "Muscle Stretching Exercises" OR "Electrolysis"))

Scielo, WOS Core Collection (Web of Science)

TS=(("Patellar Ligament" OR "Patellar tendinopathy") AND "Exercise Therapy" AND ("Musculoskeletal Manipulations" OR ("Acupuncture Therapy" OR "dry needling") OR ("Muscle Stretching Exercises" OR stretching)OR ("Electrolysis" OR EPI OR "intratissue percutaneous electrolysis")))

Pedro

Patellar tendinopathy AND exercise AND Musculoskeletal Manipulations

Patellar tendinopathy AND exercise AND acupuncture

Patellar tendinopathy AND exercise AND stretching

Patellar tendinopathy AND exercise AND electrolysis

Google scholar

patellar tendinopathy AND soft tissue AND exercise therapy AND pain AND function AND random *
