# Supplementary material for: The Effects of Soft-Tissue Techniques and Exercise in the Treatment of Patellar Tendinopathy—Systematic Review and Meta-Analysis
Source: Healthcare (Basel). 2024 Feb 7;12(4):427. doi: 10.3390/healthcare12040427 (PMC10887760; doi:10.3390/healthcare12040427)
Supplement: Supplementary file 1 [file healthcare-12-00427-s001.zip › healthcare-2730263-supplementary S2.pdf]

Supplementary Material S2: Characteristics of the intervention

Author	Groups	Intervention	Structure	Intensity	Reps/sets	Frequency	Duration
Dimitrios et al. 2012	ET+ES	● Static stretching<br>● Single-leg squat on a 25° inclined plane	Quadriceps muscle and patellar tendon.	No pain, little pain	● 30" 1' rest<br>● 3x15 2' rest	5 times/wk	4 weeks
	GC	● Single-leg squat on a 25° inclined plane		No pain, little pain	● 3x15 2' rest		
Dragoo et al. 2014	DN	● Eco-guided dry needling<br>● 5-phase eccentric exercise program	Quadriceps muscle and patellar tendon.	n/m	● 10 punctures<br>● n/m	n/m<br>2 times/wk	12 weeks
	GC*	● 5-phase eccentric exercise program			n/m	2 times/wk	
Jadhav et al. 2014	MFT	● Transverse friction massage<br>● Quadriceps and hamstring stretching<br>● Bilateral squat with 25° inclined plane	Quadriceps muscle and patellar tendon.	n/m	● 10’<br>● 3x20"<br>● 3x15	5 times/wk	12 weeks
	GC	● Cryotherapy<br>● Quadriceps and hamstring stretching<br>● Bipodal squat with a 25° inclined plane			● 10’<br>● 3x20"<br>● 3x15	5 times/wk	

Author	Groups	Intervention	Structure	Intensity	Reps/sets	Frequency	Duration
Abat et al. 2016	EPI	• Eco-guided EPI<br>• Single-leg squat on a 25° inclined plane	Quadriceps muscle and patellar tendon.	2 mA<br>n/m	• 3 punctures<br>• 3x15,3' rest	every 2 weeks<br>3 times/wk	8 weeks
	GC**	• Single-leg squat on a 25° inclined plane		n/m	• 3x15,3' rest	3 times/wk	
López-Royo et al. 2021	DN	• Eco-guided dry needling<br>• Single-leg squat on a 25° inclined plane	Quadriceps muscle and patellar tendon.	n/m<br>pain 5/10	• 3 punctures of 3"<br>• 3x15	every 2 weeks<br>2 times a day	8 weeks
	EPI	• Eco-guided EPI<br>• Single-leg squat on a 25° inclined plane		3mA<br>pain 5/10	• 1 punctures of 3"<br>• 3x15	every 2 weeks<br>2 times a day	
	GC	• Single-leg squat on a 25° inclined plane		pain 5/10	• 3x15	2 times a day	
Sharif et al. 2023	DN	• Eco-guided dry needling<br>• Bilateral and unilateral squat with 25° inclined plane plus conventional physiotherapy.	Quadriceps muscle and patellar tendon.	No pain, little pain	• 20-30 punctures of 3"<br>• 3x15	2 times/wk	4 weeks
	GC	• Bilateral and unilateral squat with 25° inclined plane plus conventional physiotherapy.			• 3x15		

DN: Dry needling; **EPI**: Percutaneous Electrolysis Guided by Ultrasound; **ES**: Stretching; **ET**: Therapeutic Exercise; **GC**: Control Group; **MFT**: transverse friction massage; **n/m**: not mentioned; *6 ml of leukocyte-rich PRP was also applied; **ultrasound, laser, and interferential current were also applied.
